# A Case Report on the Development of Ulcerative Colitis During Osimertinib Therapy for Epidermal Growth Factor Receptor Mutation‐Positive Non‐Small‐Cell Lung Cancer

**DOI:** 10.1002/rcr2.70272

**Published:** 2025-07-10

**Authors:** Tomoki Tamura, Misaki Tani, Hiromichi Ogata, Tomoya Osedo, Masahiro Yamashita, Taisaku Koyanagi, Tatsuya Nishi, Takahiro Umeno, Shoichi Kuyama

**Affiliations:** ^1^ Department of Respiratory Medicine NHO Iwakuni Clinical Center Yamaguchi Japan

**Keywords:** epidermal growth factor receptor, non‐small cell lung cancer, osimertinib, ulcerative colitis

## Abstract

Osimertinib, a standard treatment for epidermal growth factor receptor (EGFR)‐positive non‐small‐cell lung cancer, commonly causes manageable diarrhoea. We report osimertinib‐induced refractory diarrhoea diagnosed as ulcerative colitis, remitting with mesalazine. A 69‐year‐old woman with advanced‐stage EGFR mutation‐positive lung adenocarcinoma developed persistent diarrhoea 3 weeks after starting osimertinib, complicated by anorexia, hypotension, anaemia and renal failure requiring hospitalisation. While anaemia and renal failure improved after osimertinib cessation, diarrhoea persisted. A colonoscopy revealed ulcerative colitis, confirmed by neutrophil and lymphocyte infiltration in the intestinal mucosa, glandular deformation and decreased goblet cells. This case demonstrates that osimertinib‐induced ulcerative colitis was successfully treated with mesalazine.

## Graphical abstract

In general, diarrhoea induced by osimertinib improves with symptomatic treatment and withdrawal. We diagnosed ulcerative colitis via colonoscopy in a patient with osimertinib‐induced diarrhoea that did not respond to treatment.

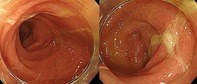

## 1 Introduction

Osimertinib, a third‐generation epidermal growth factor receptor (EGFR)‐tyrosine kinase inhibitor (TKI), is the standard of care for EGFR mutation‐positive non‐small cell lung cancer (NSCLC). It commonly causes diarrhoea, which is usually managed with diarrhoea management, dose reduction or discontinuation. While two cases of osimertinib‐induced ulcerative colitis were reported in a Japanese post‐marketing surveillance, we present a case of refractory diarrhoea after starting osimertinib, diagnosed as ulcerative colitis and successfully remitted with mesalazine (5‐aminosalicylic acid).

## 2 Case Report

**FIGURE 1:**
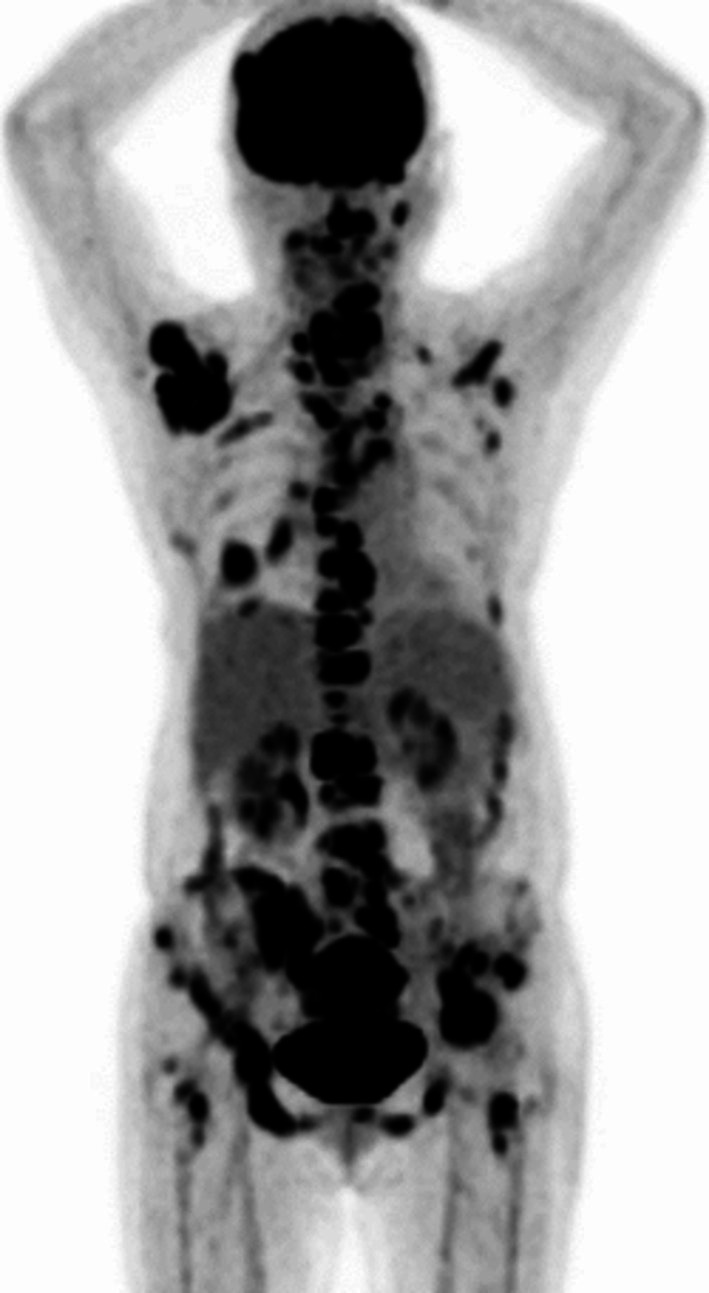
Positron emission tomography‐computed tomography examination showing fluorodeoxyglucose accumulation in the right lung mass, lymph nodes, bone and thyroid gland.

**FIGURE 2:**
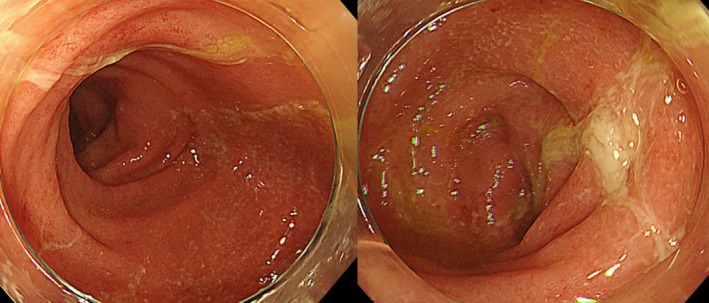
Colonoscopy showing continuous mucosal oedema from the sigmoid colon to the rectum, decreased vascular translucency, mild erythema and adhesion of purulent secretions.

**FIGURE 3:**
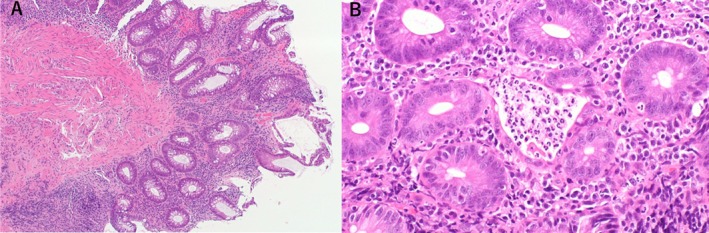
Histological diagnosis revealed a strong infiltrate of neutrophils and lymphocytes in the intestinal mucosa, with areas of glandular deformity, decreased goblet cell count, and a crypt abscess (Haematoxylin and Eosin staining A: ×40, B: ×200).

A 69‐year‐old woman diagnosed with stage IVB (cT2aN2M1c) EGFR exon 21 mutation‐positive lung adenocarcinoma with multiple bone and thyroid metastases (Figure 1) and Eastern Cooperative Oncology Group (ECOG) performance status 2. The patient received radiation therapy for multiple bone metastases and was treated with osimertinib. She initially developed mild diarrhoea, responsive to antidiarrheals. However, 3 weeks later, diarrhoea persisted, complicated by anorexia and hypotension; 6 weeks later, she was hospitalised with renal failure and anaemia. The renal failure was prerenal in nature and improved rapidly after osimertinib was discontinued and intravenous fluids were administered. The patient had mild microcytic anaemia with normal vitamin B12, folic acid and erythropoietin levels that were elevated. The anaemia did not worsen after one blood transfusion. However, the patient's diarrhoea did not improve, even with the administration of antidiarrheal medication. A colonoscopy performed on the third day of hospitalisation revealed ulcerative colitis from the sigmoid colon to the rectum, characterised by mucosal oedema, decreased vascular translucency, purulent secretions, erythema and erosion (Figure 2). *Clostridioides difficile*‐associated enteritis, cytomegalovirus infection, and Epstein–Barr virus infections were ruled out based on histological diagnosis, bacterial culture, and serological tests. Histological analysis confirmed ulcerative colitis, showing neutrophil and lymphocyte infiltration in the intestinal mucosa and areas showing glandular deformation and decreased or hypodense goblet cell counts (Figure 3). The patient was given mesalazine at a dose of 4000 mg per day. Three days after starting the medication, the patient had a bowel movement with formed stool. After a three‐week break, the patient resumed osimertinib at a reduced dose of 40 mg while continuing mesalazine and the patient was discharged with stable bowel function. The patient has since continued osimertinib therapy.

## 3 Discussion

Osimertinib is a third‐generation EGFR‐TKI, a first‐line treatment for EGFR mutation‐positive NSCLC and T790M mutated NSCLC resistant to first‐ and second‐generation EGFR‐TKI. While diarrhoea is a common side effect of osimertinib, severe diarrhoea is rare.

**FIGURE 4:**
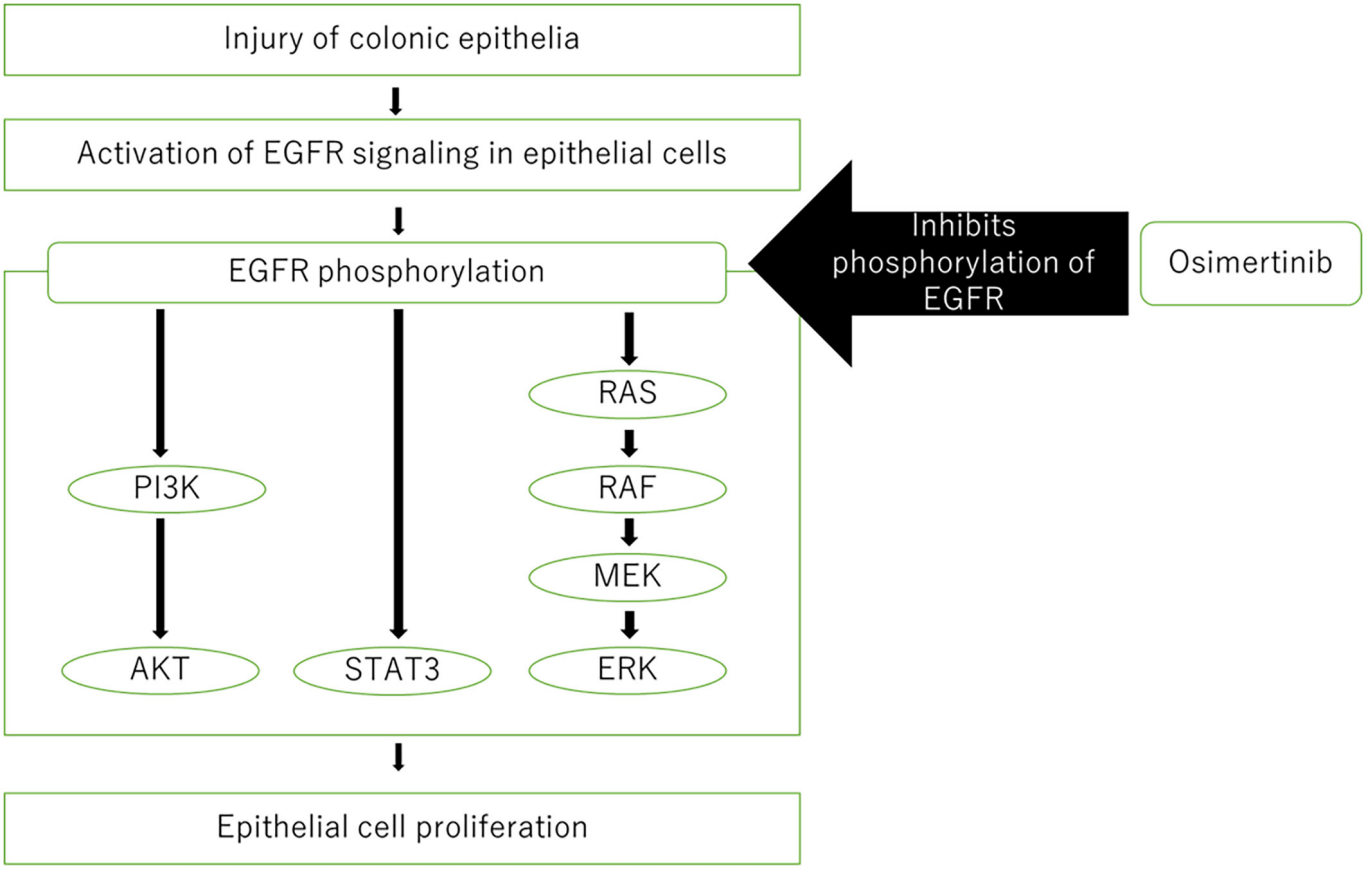
Shema of the EGFR signalling pathway in intestinal epithelial regeneration, where phosphorylation of EGFR triggers activation of the PI3K, STAT3 and MAPK pathways, thereby causing epithelial regeneration. MAPK, mitogen‐activated protein kinase; PI3K, phosphatidylinositol 3‐kinase; STAT3, signal transducer and activator of transcription 3.

We report a case of osimertinib‐induced ulcerative colitis. Although osimertinib‐induced colitis and relapse of anti‐PD‐1‐related enteritis with osimertinib have been reported, cases of ulcerative colitis specifically have not been documented. Other agents have been reported to cause ulcerative colitis with bevacizumab for NSCLC [1]. Gefitinib, a first‐generation EGFR‐TKI, has been shown to exacerbate colitis in a mouse model of induced colitis [2]. Epithelial regeneration is an important process for recovery in the intestinal mucosa of ulcerative colitis, and EGFR stimulation is known to promote epithelial regeneration in a mouse model [3]. In intestinal epithelial regeneration, EGFR phosphorylation and activation of downstream signals phosphatidylinositol 3‐kinase, signal transducer and activator of transcription 3, and mitogen‐activated protein kinase trigger epithelial cell regeneration. The inhibition of EGFR phosphorylation by EGFR‐TKI is thought to exacerbate enteritis (Figure 4). In contrast, clinical reports suggest that intravenous epidermal growth factor (EGF) enema can improve left‐sided semicolonic ulcerative colitis [4]. It has also been shown that activation of EGFR signalling and related cell behaviours repairs damage to intercellular tight junctions and improves ulcerative colitis in a dextran sulphate sodium‐induced ulcerative colitis model [5]. Osimertinib‐induced inhibition of EGFR in the intestinal tract may exacerbate latent ulcerative colitis, triggering its clinical manifestation. To our knowledge, two cases of ulcerative colitis caused by osimertinib have been reported in post‐marketing surveillance in Japan. However, this is the first report describing the details, highlighting the need for further case reports to better understand this potential association. In conclusion, this case report describes a patient with osimertinib‐induced ulcerative colitis, successfully managed with mesalazine, suggesting a possible association between the EGFR inhibitor and ulcerative colitis exacerbation.

## Author Contributions

T.T. wrote the manuscript. All authors contributed to the manuscript editing and approved the final version of the manuscript.

## Ethics Statement

This study was approved for publication by the relevant institutional review board (no. 0653) of the NHO Iwakuni Clinical Center Institutional Review Board, Iwakuni, Yamaguchi, Japan.

## Consent

The authors declare that written informed consent was obtained for the publication of this manuscript and accompanying images using the form provided by the Journal.

## Conflicts of Interest

The authors declare no conflicts of interest.

## Data Availability

Research data are not shared.
